# Neural mechanisms for learning self and other ownership

**DOI:** 10.1038/s41467-018-07231-9

**Published:** 2018-11-12

**Authors:** Patricia L. Lockwood, Marco K. Wittmann, Matthew A. J. Apps, Miriam C. Klein-Flügge, Molly J. Crockett, Glyn W. Humphreys, Matthew F. S. Rushworth

**Affiliations:** 1 Department of Experimental Psychology, University of Oxford, Oxford, OX1 3PH UK; 2 Wellcome Centre for Integrative Neuroimaging, Department of Experimental Psychology, University of Oxford, Oxford, UK; 3 Department of Psychology, Yale University, New Haven, CT 06511 USA

## Abstract

Sense of ownership is a ubiquitous and fundamental aspect of human cognition. Here we used model-based functional magnetic resonance imaging and a novel minimal ownership paradigm to probe the behavioural and neural mechanisms underpinning ownership acquisition for ourselves, friends and strangers. We find a self-ownership bias at multiple levels of behaviour from initial preferences to reaction times and computational learning rates. Ventromedial prefrontal cortex (vmPFC) and anterior cingulate sulcus (ACCs) responded more to self vs. stranger associations, but despite a pervasive neural bias to track self-ownership, no brain area tracked self-ownership exclusively. However, ACC gyrus (ACCg) specifically coded ownership prediction errors for strangers and ownership associative strength for friends and strangers but not for self. Core neural mechanisms for associative learning are biased to learn in reference to self but also engaged when learning in reference to others. In contrast, ACC gyrus exhibits specialization for learning about others.

## Abstract (web summary)

The sense of ownership – of which objects belong to us and which to others - is an important part of our lives, but how the brain keeps track of ownership is poorly understood. Here, the authors show that specific brain areas are involved in ownership acquisition for the self, friends, and strangers.

## Introduction

Sense of ownership is such a fundamental aspect of human cognition that it influences the grammar of many human languages. For example, words are changed and used in special ways such as the genitive case or the construct state to indicate ownership associations. Psychological ownership—the feeling of ownership over objects both material and immaterial that develops over time^1^—is distinct from legal ownership in terms of malleability, responsibility and genesis^1^. Several studies suggest that acquiring a sense of ownership can profoundly change our perception, memory, attention and decision-making^2–8^. It has long been thought, since the time of William James, that ownership may be underpinned by associative processes^9^ and it is this possibility that we examine empirically here.

Associating shapes with oneself alters perceptual processing, making people faster and more accurate than when the same associations are formed with other people (reviewed by Sui and Humphreys^7^). Memory recall is enhanced when stimuli are categorized in relation to oneself than to others^3,4,10,11^. People put in more effort to help themselves than others^12^ and learning rates are higher when learning about one’s own reward outcomes than another person’s^13^. At a basic level, ownership of an item increases its value and desirability, as shown in economic studies of the endowment effect^6^ and in social psychological studies of mere ownership^2,5^. Together, these studies suggest that the processing of information related to ourselves is facilitated, such that it is better recalled, processed more rapidly, and biases our preferences, learning and decision-making, compared to information related to other people. This ‘self-ownership bias’ could therefore have important implications for studying learning, decision-making and social cognition. However, how ownership associations are formed, and the computational and neural mechanisms that underpin them are poorly understood.

Associative learning theory provides a powerful theoretical framework to understand the behavioural and neural basis of how we acquire associations between stimuli and responses in social and non-social contexts^14,15^. In this framework, stimulus–response outcome associations are updated over time by prediction errors that signal the discrepancy between expectations and outcomes^15,16^. Can the sense of ownership be understood using the same associative processes assumed to underlie reward-based learning? The ventromedial prefrontal cortex (vmPFC) has been consistently linked to associative value representations at the time of choice, while the ventral striatum is linked to prediction error signals at the time of an outcome^15,17–22^.

Intriguingly, the same area, vmPFC and adjacent medial prefrontal cortex (mPFC), including areas 14 and 11 m^23,24^, have been linked to the processing of self-relevant information^7,8,25–27^. Several studies have reported greater responses in this area when processing self-relevant as opposed to other-relevant information, suggesting that vmPFC is specifically concerned with processing the self (reviewed in ref. ^7^). However, vmPFC has also been linked to simulated reward associations for self and other^28^, as well as value-based choice and value difference^19,29–33^. Therefore, an alternative hypothesis is possible: social information is processed in reference to oneself, and vmPFC should reflect ownership associations relating not just to self but also to others. By using a parametric design, it is possible to examine whether vmPFC activity in relation to ownership is the same or distinct for different types of ownership association. Finally, there is evidence that mPFC may reflect self and other relevant information in a spatial gradient with self-related activity increasingly prominent as one moves towards vmPFC and other related processing increasingly prominent as one moves towards dorsal–medial PFC (dmPFC)^34^.

Other studies have suggested brain regions in which social information may be preferentially processed. In particular, dorsal portions of the mPFC, such as area 9, have been linked to processing information specifically about others^35^, during mentalizing^36^ and tracking others’ performance^37^. The anterior and mid-cingulate contains several sub-regions. Some parts of the gyrus of the dorsal anterior and mid-cingulate cortex may be particularly concerned with social learning^16,35,38–44^, whereas a more dorsal region in the anterior cingulate cortex (ACC) sulcus has a general role in learning and decision making in relation to both self and others^35,39,41,42,44^. Following previous authors^16,39,41,42,44^, we refer to these two subregions in the sulcus and gyrus as ACCs and ACCg, respectively. By utilizing an associative learning framework to probe agent–object associations over time, we might be able to understand the roles of these different regions in self and other ownership acquisition.

**Fig. 1:**
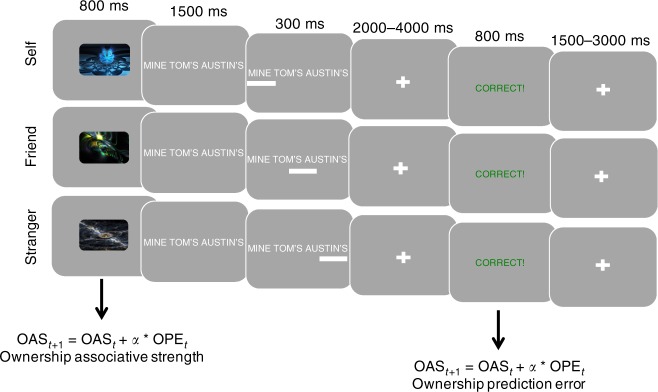
Associative learning of self and other ownership. Participants performed an associative learning task where they learnt about pictures that belonged to themselves, their best friend, or to a stranger. Participants were presented with a fractal image and learnt by trial and error the ownership of the different fractals. We used an associative learning model to calculate parametric values of ownership associative strength (OAS) between picture and label at the time of the picture (the strength of ownership) and the size of the ownership prediction error (OPE) at the time of the outcome. Each fractal image was presented ten times in a pseudorandom order. There were eight pictures per each agent presented over two blocks to encourage learning while minimizing working memory load

Here we employ associative learning models to examine the emergence of self and other ownership. We use a ‘minimal ownership’ paradigm: participants repeatedly encounter abstract pictures and learn whether they belong to themselves or to a close friend or a stranger (Fig. 1). We used two types of other agents because there is evidence that different neural mechanisms are engaged when processing others based on similarity to the self^45^. This paradigm, inspired by social psychological studies of minimal groups (reviewed in ref. ^46^), has the important advantage of controlling for previous stimuli associations. We could also theoretically match irrelevant features such as familiarity of the different stimuli.

Importantly, ownership entails an association between an agent (self, friend, stranger) and an object^9^, which in itself has no intrinsic reward value. We therefore designed a paradigm where ownership did not entail receipt of reward. We fit an associative learning model to behaviour that allowed us to probe different learning rates, brain regions that track ownership associative strength (OAS) at the time of choice, and regions that track ownership prediction errors (OPEs) at the time of feedback.

We find a bias to form ownership associations in relation to oneself as opposed to others. We refer to this as a 'self-ownership bias' and it occurs at multiple levels of behaviour, from initial preferences through to reaction times and computationally defined learning rates. Several areas within medial frontal cortex track OAS between objects and all agents, with an increased response in vmPFC and ACCs to self vs. stranger. However, no brain area only tracks self-relevant information. In contrast, tracking of information for others has a distinct neural correlate; ACCg signals OPEs for strangers and OAS for strangers and friends but not oneself. Therefore, most neural mechanisms for associative learning are biased to learn in reference to oneself, but are also engaged when learning in reference to others. In contrast, ACCg is specialized for learning about other people.

## Results

### Self-ownership bias at multiple levels of behaviour

**Fig. 2:**
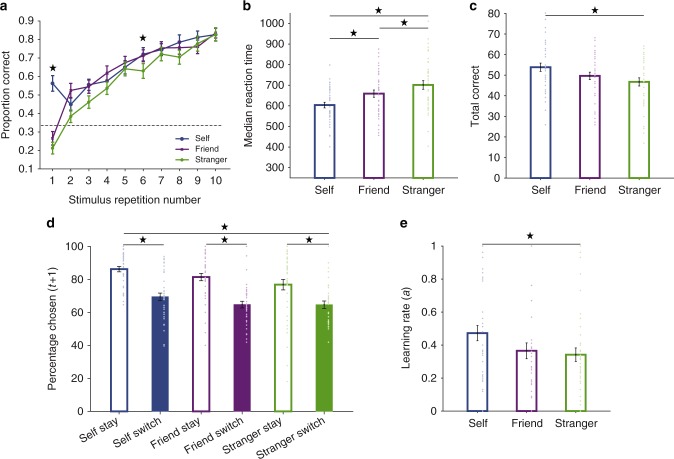
Self-ownership bias at multiple levels of behaviour. **a** Learning curve showing the average proportion correct across all participants, averaging across all stimuli. Each stimulus was presented 10 times. A significant main effect of stimulus repetition (*p* < .05) showed that participants were able to learn the stimulus–agent associations across the experimental session. However, a significant interaction between stimulus repetition×agent (with participants more correct on the first trial for self compared to friend or stranger) indicated a self-bias—a bias to label pictures as belonging to themselves with no prior information (ANOVA *p* < .05). Note that interleaved at the beginning of the experiment with the stimuli that participants could learn about were six stimuli that were presented only once (and could not therefore be learned: see Methods section). This figure does not include the six extra fractals presented only once (see Methods section). Dotted line shows chance performance level (33%). **b** Participants had faster reaction times for correct responses to self compared to friend or stranger (all conditions significantly different *p* <.05). **c** Participants were overall more correct at assigning ownership to self pictures than to friend or stranger pictures (self significantly different from both friend and stranger, *p* < .05). **d** Learning behaviour showed evidence of both a self-bias and confirmation bias with participants more likely to correctly stay and correctly switch after a self choice compared to friend and stranger (main effect of agent, *p* < .05) and to learn better from correct than incorrect information (main effect of stay/switch, *p* < .05). Empty bars indicate positive and shaded bars negative feedback on trial *t*. X-ticklabels indicate correct stay/switch behaviour after agent-specific feedback. **e** Learning behaviour also showed a self prioritization with higher learning rates for self compared to stranger (*p* < .05). Asterisks indicate significant difference at *p* < .05. Error bars represent standard error of the mean. All *n* = 39

We first examined whether there was a self-ownership bias in how people responded to and learnt about associations between agents and objects. We observed such a bias at multiple levels of behaviour (Fig. 2). People were more likely to say that a picture belonged to themselves than to another person on the first presentation of each picture, that is, there was a tendency to indicate pictures belonged to oneself when faced with no prior knowledge of ownership (analysis of variance (ANOVA) main effect of agent (*F*(2,76) = 5.26, *p* = .007, *η*^2^ = .12), main effect of trial number (*F*(9,342) = 93.36, *p* < .001, *η*^2^ = .71) indicating significant learning over the course of the experiment, and agent ×trial number interaction (*F*(18,684) = 5.56, *p* < .001, *η*^2^ = .13). This average tendency to label pictures as ‘mine’ was therefore used as a starting value in our computational model.

We also observed that participants were quicker to respond correctly to self-stimuli (ANOVA main effect of agent (*F*(2,76) = 23.72, *p* < .001, *η*^2^ = .38; self median = 603.87, SD = 86.15; friend median = 659.34, SD = 109.90; Stranger median = 701.67, SD = 130.58, all *p* < .007) and more accurate at correctly assigning the ownership of pictures that belonged to themselves compared to others (ANOVA main effect of agent (*F*(2,76) = 7.50, *p* = .001, *η*^2^ = .17; self correct = 53.85, SD = 12.67; friend correct = 49.64, SD = 11.27; stranger correct = 46.72, SD = 12.33, *p* < .03). Next, we considered whether faster reaction times and accuracy might simply result from participants initial bias to indicate that stimuli were owned by the self. An additional control analysis (Supplementary Note 1) showed that this was not the case.

We then assessed whether there was evidence for a self-ownership bias in learning. We analysed the proportion of next trials (trial *t* + 1) on which participants correctly chose the same agent–picture association again after having received correct feedback for their choice on the current trial (trial *t*), and the proportion of trials (trial *t* + 1) on which they correctly switched away from an agent after incorrect feedback on the current trial (t). Several studies have suggested a confirmation bias in associative learning, with people learning more from correct than incorrect feedback^47–49^. We therefore tested whether there was a self-bias in learning, a confirmation bias in learning, and whether the two effects interacted. Compared with friend and stranger choice trials, we found a self-bias in learning (ANOVA main effect of agent, *F*(2,76) = 8.51, *p* < .001, *η*^2^ = .18), with a higher likelihood of repeating a correct self choice after being correct and a higher likelihood of switching away from a self choice after being incorrect (*p* = .008 compared to friend, *p* < .001 compared to stranger). There was no significant difference between friend and stranger (*p* = .228). We also found a confirmation bias with participants more likely to repeat a correct choice after positive feedback than to switch away from an incorrect choice after negative feedback (ANOVA main effect of stay/switch (*F*(1,38) = 89.06, *p* < .001, *η*^2^ = .70)). However, there was no interaction between the self-bias and confirmation bias (*F*(2,76) = .95, *p* = .393, *η*^2^ = .02) suggesting relatively independent effects of self-ownership bias and confirmation bias on decision-making.

Another way to assess whether participants have a self-ownership bias in learning is to examine whether there was a higher learning rate for self. We found a significant main effect of agent on learning rates (ANOVA *F*(2,76) = 3.14, *p* < .05, *η*^2^ = .08), with a higher learning rate for self-picture associations (*M* = .47, *SD* = .29) than stranger–picture associations (*M* = .34, SD = .26, *p* = .017), but not friend–picture associations (*M* = .37, SD = .30, *p* = .097). There was no significant difference between agents in inverse temperature (*F*(2,76) = .21, *p* > .80, *η*^2^ = .006).

Our model of learning employs gradual changes in OAS. We took this approach because such models frequently provide good accounts of learning even when associations are deterministic (either right or wrong)^50^. Nevertheless, we performed a final set of behavioural analyses to examine whether an alternative deterministic learning model provided a better account of our data and also a model that treated the initial self bias as a free parameter. The original model provided the better account of participant behaviour compared to a number of other alternative models. (Supplementary Figure 1, Supplementary Table 1 and Supplementary Note 2).

### Common coding of ownership

For the trial-by-trial tracking of OAS and OPE, we first examined areas that commonly coded ownership associations for all three agents by performing conjunction analyses (this is possible because of the statistical independence of the key regressors; Supplementary Figure 2). All results are reported at whole-brain family-wise error (FWE)-corrected or small-volume corrected (SVC) using a combined anatomical mask of several mPFC regions (see Methods section). We tested whether areas typically activated in studies of reward associative learning, particularly ventral striatum, responded to OPEs.

Several areas of the mPFC and temporoparietal junction (TPJ) commonly tracked OAS for all agents (see Supplementary Table 2). Importantly, these regions included area 8/9 in the dmPFC and the TPJ, areas consistently linked to social processing^35,51^. However, these regions tracked OAS for self and other, rather than signalling self exclusively^7,8^ or other-related information^36^ as suggested in previous studies.

**Fig. 3:**
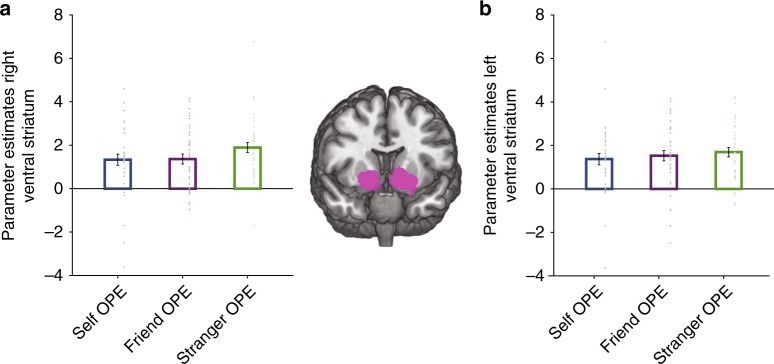
Common coding of ownership prediction errors (OPEs) for all agents in ventral striatum. Ventral striatum signalled prediction errors for all agents (*p* < .001, FWE-whole brain corrected). **a** Right ventral striatum. **b** Left ventral striatum. Activation overlaid on an anatomical scan of the coronal surface. Error bars indicate SEM. *N* = 39

Consistent with several studies of associative learning^13,15,16,22,52^, the bilateral ventral striatum (Left: *x* = −14, *y* = 10, *z* = −8; *Z* = 7.23, *k* = 959, *p* < .001 FWE-whole brain and Right: *x* = 16, *y* = 6, *z* = −12; *Z* = 6.37, *k* = 554, *p* < 0.001 FWE-whole brain, Fig. 3) signalled prediction errors in all three learning conditions (see Supplementary Table 2). We also found responses in the superior frontal gyrus in the vicinity of area 8 m (ref. ^24^; *x* = −18, *y* = 30, *z* = 56; *Z* = 4.73, *k* = 404, *p* = .029 FWE-whole brain). Activity in area 9 in the dmPFC-coded OPEs for all agents (*x* = −6, *y* = 66, *z* = 16; *Z* = 4.13, *k* = 24, *p* < .05 FWE-SVC). No other brain area significantly tracked OPEs in all learning conditions.

### Ownership by the self and vmPFC

**Fig. 4:**
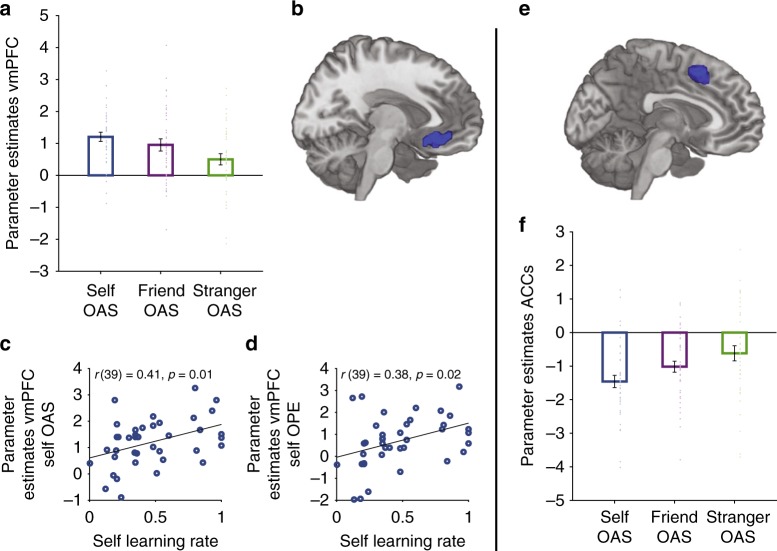
vmPFC and ACCs track ownership associative strength (OAS) for self more than stranger, but carry signals of all three agents. **a** vmPFC (area 14 m extending to 11 m) signals covary with OAS for self more than stranger (*p* < .05 FWE-SVC), but respond significantly from 0 in each of the three conditions (all *p*s < .008). **b** Cluster overlaid on an anatomical scan of the medial surface. **c** Bivariate correlations between vmPFC responses to self OAS and the self learning rate and **d** correlations between vmPFC responses to self OPEs and self learning rate. In both cases, correlations with the self learning rates and neural responses were significantly stronger than associations between vmPFC responses to friend and stranger and respective learning rates (during the tracking of OAS) and vmPFC responses to stranger and stranger learning rates (during the tracking of OPEs). **e** Cluster overlaid on an anatomical scan of the medial surface. **f** Negative ACC signals covary with OAS for self more than stranger (*p* < .05 FWE-whole brain), but respond significantly differently from 0 in each of the three conditions (all *p*s < .010). Error bars indicate standard error of the mean. *N* = 39

To identify neural responses that underpin a self-ownership effect, we next examined neural responses that tracked OAS between agent and object for self more than stranger. For all analyses of specific coding, we focused on linear contrasts of agent (1 0 −1 and −1 0 1) corresponding to the self, friend, stranger conditions. We found that only the vmPFC showed stronger tracking of self compared to stranger (area 14 m extending into area 11 m; MNI coordinates: *x* = −6, *y* = 28, *z* = −14; *Z* = 3.91, *k* = 212, *p* < .05 and *x* = 6, *y* = 22, *z* = −14, *p* < .05 voxel-level SVC-FWE, Fig. 4a, b). Intriguingly, however, although vmPFC responded to self OAS more than stranger OAS, it tracked ownership information about all three agents, with responses to OAS significantly above 0 in all three learning conditions (all *p*s < .008) (Fig. 4). No other brain area showed this pattern. Response in the vmPFC also remained unchanged even after controlling for trial-by-trial reaction time (see Methods), suggesting that differences in activity in this area for self compared to stranger did not simply reflect the reaction time differences between conditions. We also re-ran all analyses using parametric values from a model with a single, rather than separate, learning rates and found the same results.

As mentioned previously, vmPFC has been argued, on the one hand, to mediate a self-bias effect^7,8,26^ but, on the other hand, associated with decision signals more generally^18,21,31,32,53,54^. We therefore tested whether learning rates in the three conditions were correlated with responses in vmPFC to understand whether increased vmPFC tracking of self vs. stranger-related OAS simply reflected a higher learning rate for self compared to other agents or a self-ownership bias independent from learning rate. Note that this analysis is statistically orthogonal to the whole-brain contrast used to define the cluster.

As hypothesized, we found a significant correlation between vmPFC responses to self-OAS and the self learning rate (*r*(39) = .41, *p* = .01, 95% confidence interval (CI): .11, .64, Fig. 4c). Intriguingly, however, there was no significant correlation between vmPFC responses to friend and friend learning rate (*r*(39) = .00 *p* = .99, 95% CI: −.32, .32) or between vmPFC responses to stranger and stranger learning rate (*r*(39) = .070, *p* = .68, 95% CI: −.25, .38). Moreover, the correlation between self learning rate and self OAS tracking in vmPFC was significantly stronger than associations with the other agents (see Supplementary Note 3). We next tested whether average responses in this independent region of interest (ROI) from the OAS period also tracked OPEs at the time of feedback and found significant average effects for all learning conditions (see Supplementary Note 3). We also observed significant correlations with the learning rate for self and self vmPFC responses to OPEs (Fig. 4d), and again these were significantly higher than stranger responses, but not friend responses (see Supplementary Note 3). Finally, we also examined whether there was any evidence for a spatial gradient of ownership spanning ventral to dorsal portions of mPFC, as suggested in previous studies^34,55^. However, we found no evidence for a gradient reflecting self/other, but we did observe a gradient of stranger OAS from ventral (weakest) to dorsal (strongest) parts of mPFC (see Supplementary Figure 3 and Supplementary Note 4 for full details of analyses and statistics).

Together, these findings suggest that the self-ownership bias in associative learning is reflected in neural signals tracking both OAS at the time of decision making and OPE at the time of feedback. The difference was most clear when ownership relating to the self was compared with ownership relating to other agents that were most different to the self, that is, strangers, as opposed to other agents that were perceived as similar to the self, such as friends.

### Ownership by the self and ACCs

We next examined the reverse contrast for areas encoding stranger OAS more than self OAS. We identified responses at the whole-brain level in the ACCs and adjacent mPFC (*x* = −8, *y* = 12, *z* = 48; *Z* = 5.57, *k* = 318, *p* < .001 whole-brain FWE-corrected, Fig. 4e, f). Initially this might be taken as evidence of a relative specialization for other-related processing in ACCs. However, on closer inspection activity in this area in fact simply had a negative relation with the OAS of the choice taken, as has previously been reported even in many other situations including non-social tasks^56–58^.

The explanation for this negative coding pattern has been debated elsewhere^57–59^. In the present context, the negative coding scheme in ACCs and adjacent mPFC suggests that rather than concluding that the activity tracked other-referenced associations more strongly than self-referenced ones it seemed more parsimonious to conclude that it negatively tracked OAS for all three agents. Moreover, as in the vmPFC, the coding pattern was present for all three agents, self, friend and stranger, with the pattern for self stronger than for other agents that were strangers (all *p*s < .010) (Fig. 4c). However, unlike with vmPFC, we did not observe any significant correlations between learning rates and ACCs. Moreover, as for vmPFC, including reaction time in the generalized linear model (GLM) did not change this result. No other brain area showed this pattern.

### Specific ownership learning signals in ACCg for others

**Fig. 5:**
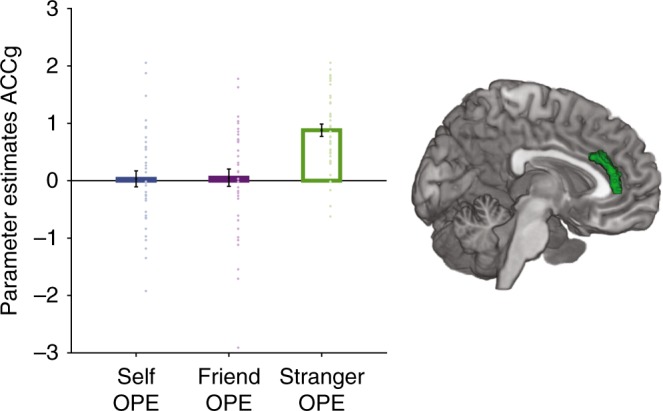
Specific coding of ownership prediction errors (OPEs) for strangers in ACCg. ACCg (area 24) specifically coded ownership prediction errors when learning about the ownership of strangers (*p* < .03, FWE-SVC for an anatomical mask of the medial prefrontal cortex). Activation overlaid on an anatomical scan of the medial surface. Error bars indicate standard error of the mean. *N* = 39

The ACCg, a dorsal cingulate region that lies dorsal to the callosum but ventral to the ACCs in Fig. 4c has been linked to the encoding of social information^39,42,44^. We therefore took special care to investigate activity in the anatomical region (area 24) described by Neubert et al.^24^ in which such effects have been reported. We observed OPEs that were specifically related to learning about strangers in ACCg (*x* = 10, *y* = 38, *z* = 6; *Z* = 4.33, *p* < .02 and *x* = 6, *y* = 30;  *z* = 28; *Z* = 4.25, *p* < .03, *k* = 202, FWE-SVC) (see Fig. 5 and Supplementary Figure 4)). We used this specific cluster of ACCg to test whether OAS signals when learning about strangers were also coded in ACCg by taking the average parameter estimates over the whole ROI. We found that there was also a significant response in ACCg that tracked stranger OAS (*t*(38) = 1.95, *p* = .03). Further analysis of the same ROI found that while ACCg also tracked friend OAS (*t*(38) = 2.09, *p* = .02), it did not track self OAS (*t*(38) = .93, *p* = .18). We also conducted a reverse inference meta-analysis using Neurosynth^60^ combining masks of *n* = 1220 studies linked to keywords of ‘value’ and ‘learning’ to test whether this ACCg area is commonly seen in studies of learning and value in non-social settings. We found no significant responses in ACCg that overlapped with domain-general areas responding to value and learning (see Supplementary Note 5, Supplementary Figure 5 and Supplementary Figure 6).

In summary, while we had found that it was incorrect to characterize vmPFC as specialized solely for self-related associations, ACCg does appear relatively specialized for information relating to agents other than the self. OPE signals for strangers were specifically tracked in ACCg, and OAS was significantly represented for both friends and strangers but not for self. These findings point to a key role for ACCg in social learning.

**Fig. 6:**
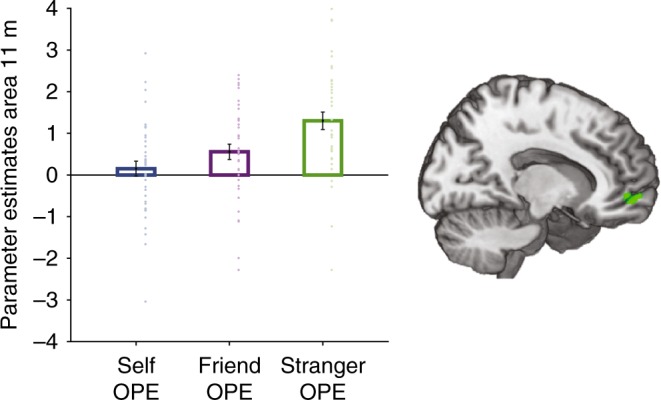
Distinct coding of ownership prediction errors in area 11 m. Dorsal parts of area 11 m (*x* = 10, *y* = 50, *z* = −6; *Z* = 3.99, *k* = 83, *p* < .04 FWE-SVC) signalled prediction errors for stranger more than self but in fact tracked prediction errors for both friend (*p* = .004) and stranger (*p* < .001) and not self (*p* > .05). Activation overlaid on an anatomical scan of the medial surface. Error bars indicate standard error of the mean. *N* = 39

Because we had found evidence for relative specialization in ACCg for OAS for agents other than the self, we also re-examined activity in the vmPFC for evidence of other related processing specialization (contrast −1 0 1). We found a smaller cluster surviving SVC in dorsal parts of area 11 m (*x* = 10, *y* = 50, *z* = −6; *Z* = 3.99, *k* = 83, *p* < .04 FWE-SVC, Fig. 6) that responded to stranger more than self, but in fact tracked prediction errors for both friend (*p* = .004) and stranger (*p* < .001). At the whole-brain level, we also found stranger-specific OPEs in posterior cingulate cortex (*x* = −10, *y* = −26, *z* = 32; *Z* = 5.19, *k* = 692, *p* < .01 FWE-whole brain) and a ventral portion of the mid insula (BA 13; *x* = −36, *y* = 8, *z* = −12; *Z* = 4.61, *k* = 104, *p* < .05 FWE-whole brain). No other brain area showed this pattern.

In summary, vmPFC and ACCs have a fundamental role in tracking OAS. While not specialized for self-related processing, these regions are biased to code information in regard to the self. Moreover, the rate at which people formed ownership associations between their own selves and objects is most strongly tracked in vmPFC. By contrast, adjacent to both these well-studied regions, most notably in ACCg but to a lesser extent in dorsal 11 m in vmPFC, we found evidence for specialized areas coding for a learning-related signal, prediction errors, in relation to other agents.

## Discussion

It has long been thought that acquiring a sense of ownership over an object requires an association between the object and the self^9^. Multiple studies have shown how this sense of ownership pervades our perception, attention, memory, learning and decision-making^5–7^, but the associative mechanisms mediating the link between ourselves, others and ownership are unknown.

We found evidence for a self-ownership bias at multiple levels of behaviour. Participants had an initial preference to say that pictures belonged to themselves, when theoretically they should have designated ownership with equal probability to all three possible agents. They were faster to respond and more accurate when learning about self-related objects than objects linked to other people, and they formed representations between themselves and objects more rapidly as indexed by a higher learning rate when acquiring self-referenced associations.

We were, however, unable to find evidence of a brain region solely concerned with the forging of associations with the self. Instead, we found evidence that two cortical regions, already known to have a role in learning reward outcome-related associations and reward outcome association-guided decision making, vmPFC and ACCs (and adjacent medial frontal cortex), had a broad role in learning associations with all three types of agents. Significant coding of other as well as self-related information in vmPFC is concordant with other studies showing a role for vmPFC in simulating others decisions^28^. Nevertheless, in both vmPFC and ACCs, there was a bias towards coding object associations in relation to the self. For example, in vmPFC self-related activity was stronger that other-related activity (Fig. 4a) and individual variation in self-related activity was correlated with individual variation in self-learning rates (Fig. 4c, d). The association between learning rates for self and vmPFC was also significantly stronger than association between vmPFC signals for other agents and learning rates for other agents. Therefore, correlations between behaviour and vmPFC response did not simply reflect a higher learning rate for self but also a self-ownership bias in behaviour. Our findings highlight the vmPFC as fundamental for self-related processing by showing that vmPFC is biased to learn in reference to oneself. However, by using a parametric design we show that although this area is biased to learn in reference to oneself, vmPFC also significantly tracks information about the ownership of other agents. Our finding could have important implications for understanding the role of vmPFC in learning and decision-making, suggesting that this area may track self and other associations even outside of the context of reward-based learning.

Activity in ACCs exhibited a similar self-ownership bias. However, the pattern of activity in ACCs requires more careful explanation. On first examination ACCs appeared to be more concerned with agents other than the self; a whole-brain analysis found that ACCs activity was more negatively related to self than to friend or stranger (Fig. 4f). However, closer analysis revealed that activity in ACCs covaried negatively with the strengths of association for all three agents and that this negative activity pattern was most prominent in the case of self associations. This is consistent with a large number of studies that have found activity in ACCs negatively covarying with strength of reward association for an action that will be chosen as opposed to an action that will not currently be chosen^57,58^. The ACC pattern may reflect the evidence for making an alternative choice in a subsequent decision. Importantly, closer tracking of OAS for self compared to strangers was not associated with the trial-by-trial reaction time, suggesting that the self-bias is not explainable in terms of response selection difficulty or conflict and extends beyond a behavioural reaction time advantage when processing information about the self.

While we did not observe any brain area exclusively concerned with self-referenced associations, this is not entirely unexpected. Indeed, representations of one’s self, such as how one forms a sense of ownership over one’s own face, are highly malleable, and are readily manipulated in bodily illusions^61^. We did, however, identify an area, ACCg, that seemed particularly concerned with learning in relation to those agents that were most distinct to the self. The activity was most prominent in area 24^24^ in the supracallosal part of the cingulate cortex in what is often referred to as the dorsal ACC or dACC. However, in most non-social learning and decision-making studies, dACC activity is most prominent in the cingulate sulcus^35,52,57,58^. In the current study, we have considered the sulcal and gyral divisions of dACC separately and followed previous studies in referring to them as ACCg and ACCs^38,40,41,43^.

When humans and monkeys learn about the reward prospects of individuals other than themselves, then it is activity in ACCg that reflects these reward associations^39,42^. Similarly, activity in ACCg may reflect other non-reward-related associations, such as those mediating the link between agent and object that is central to ownership, when these associations involve agents distinct from the self. Knowledge of such other-referenced associations may be necessary for capacities such as empathy that have also been linked to ACCg^42,43,62^. In the current study, we observed OAS signals in ACCg that were related to tracking ownership by friends and strangers. We also found learning signals specifically related to others most different from the self (strangers). This dovetails with several studies reporting other-referenced reward prediction errors and value signals in ACCg^39,41,42^. However, our results suggest that even at the level of abstract ownership learning, after controlling for the receipt of reward or pain by the other agent, ACCg may show relative specialization for processing information pertaining to other agents, thus going beyond other recent work on the role of this area in social cognition^39,42^.

We also observed responses in other brain areas previously linked to socio-cognitive processing, in particular the TPJ and dmPFC^35,51,63^. Intriguingly, these areas tracked ownership associative strength for all three agents rather than specifically responding to stranger or friend and stranger like ACCg. Although these areas covaried in a domain general way with associative strength, this tracking could still reflect a process crucial for social cognition, that is, forming basic associations between agents and objects. Adjacent to dmPFC, we found that the superior frontal gyrus also showed a domain general response to all agents, but when tracking prediction error signals at the time of the outcome. The superior frontal gyrus is not often found in studies of associative learning and it would be interesting for future studies to examine further the role of this area in forming ownership associations. We did not see any responses in subgenual ACC, another area previously linked to social prediction error signalling^64^ during prosocial learning^13^ and when receiving feedback about being liked by others^65^. Moreover, we also tested for a self-other ventral–dorsal spatial gradient in mPFC as an alternative hypothesis for how self vs. other relevant information may be processed in vmPFC^34^. Our findings again suggested that self-ownership-related activity prevailed throughout much of mPFC. There was, however, some evidence that, while always weaker than self-related activity, stranger-related activity effects became stronger more dorsally in dmPFC compared to vmPFC. Our finding fits with other studies suggesting that other related information is more strongly encoded in dorsal compared to ventral–medial prefrontal areas^35,51^.

Although several studies have documented biases related to processing information that is relevant for oneself, other studies have emphasized how it is information related to other people that is preferentially processed. People will rapidly shift their preferences to closely align with those of other people^55,66,67^. When making moral decisions people are more harm aversive when considering others than themselves^68^ and the performance of others can bias how we view our own abilities^37^. One explanation for both self and other biases in information processing is that core regions for learning and decision-making, such as vmPFC and ACCs, exhibit the first bias, that is they are preferentially recruited to process self-relevant information as we have shown here. In contrast, other areas, particularly ACCg, may be somewhat specialized for processing social information and therefore have an ‘other’ associative bias. Importantly, we show that forming a sense of ownership over arbitrary fractals changes participants’ behaviour in terms of speed of responding, accuracy and learning. Future studies could examine the effects of minimal ownership on other aspects cognition and behaviour, for example, by including a post-scanning memory task or asking participants whether they would give up money to view the different fractals. Relatedly, it would be interesting for future studies to compare the effects of learning about real material objects that participants would be able to take home after the study.

Overall, we show that people have a self-ownership bias when learning about objects in the environment. They are more likely to say things belong to themselves than to others, are faster and more accurate at making self-relevant decisions and learn at a higher rate than when learning about other owned objects. While much of the brain’s learning and decision-making apparatus may track associations in relation to the self, other related information may be particularly prominent in ACCg. These findings could have important implications for learning and decision-making as well as disorders associated with aberrant ownership representations and problems with social cognition.

## Methods

### Participants

Forty right-handed healthy adults (21 females, age 19–34 years) were recruited through university participant databases. Exclusion criteria included previous or current neurological or psychiatric disorder, non-normal or non-corrected to normal vision and contraindications that prohibited magnetic resonance imaging (MRI) scanning. One participant was excluded from the analysis due to neurological abnormalities identified during scanning, leaving a final sample of 39. We conducted an a priori power calculation based on our planned sample size and desired power (80% at *α* *p* = .05) to show that with 39 subjects we had 80% power to detect a ‘medium’ effect size of *d* = 0.46 at *α* = .05 (two-tailed) in any of our behavioural measures, an effect size smaller than typically reported in this field, indicating sufficient power. All participants gave written informed consent and the study was approved by the University of Oxford Central Research Ethics Committee.

### Experimental task

Participants performed an associative-learning task where they were required to learn the picture–agent pairings (Fig. 1). The task was deterministic such that participants were told the same pictures would always belong to the same agents. There were four pictures associated with each agent (self, friend, stranger) in the first block and a further four pictures in the second block. Based on a behavioural pilot, we designed our task such that participants would not have fully learnt all stimulus–agent associations by the end of the task. This ensured that we could assess the formation of ownership associations and limited scanning time for when all associations were fully learnt. We chose not to use probabilistic picture–agent pairings in order to more realistically reflect real-life ownership where objects are either owned or not owned. Note that several studies within the framework of associative learning theory have used deterministic, rather than probabilistic, paradigms^50,69^. Indeed, classical associative learning theory^70–72^ describes how human and non-human animals learn about associations between different stimuli and responses, but is agnostic as to whether these pairings are deterministic or probabilistic. The finding that dopamine neurons encode reward prediction errors and that over time these neurons begin to respond to reward predicting cues has been made in deterministic learning regimes^50^. Subsequently, the learning of such stimulus–reward associations has been extended to account for probabilistic outcomes, but importantly testing assays still included deterministic associations (probability of outcome either 0 or 1) just as probabilistic ones^73^.

We also introduced six additional pictures that were pseudorandomly interspersed in the first mini-block and were only presented once. These pictures were introduced because a prior behavioural pilot revealed that participants had an unexpected bias to say that pictures belonged to themselves on the first trial, even though it might have been expected that they would, on average, choose each of the agent labels with equal probability (33%). These extra trials, combined with the first instance of each picture, made possible estimation of the general self-bias—the tendency to say a picture was ‘mine’—and this bias was used to model starting action values in the computational model. Responses to this single presentation of the additional images were not used in any other analysis. Therefore, there were 24 pictures in total that were each shown 10 times. To ensure that there were equal opportunities for learning the ownership of all three agents, the pictures were presented in mini-blocks of 12 stimuli so that the maximum length of time between the presentations of a particular picture was controlled. Before scanning, participants practiced learning three picture–agent pairings to familiarize themselves with the experimental task.

### Procedure

At the beginning of each trial, participants were presented with a fractal picture that was displayed for 800 ms. They were then presented with three options about whom the picture belonged to, ‘mine’ if the picture was their own, ‘friend’: the name of their best friend (changed for each subject based on their nomination of a gender-matched friend) and finally ‘stranger’: the name of a gender-matched ‘stranger’. The stranger names came from a list of names (five male, five female) that were plausible but uncommon in the United Kingdom. Before scanning participants selected a name from this list that they had no previous association with, that is, they did not know anyone with this name or the name did not make them identify a particular person. These three options, henceforth self, friend and stranger, were displayed for 1500 ms. Once selected, a bar appeared under the option to indicate that it had been chosen, for a minimum of 300 ms. This was followed by a variable delay of 2000–400 ms and then the display of an outcome of ‘correct’ or ‘incorrect’ for 800 ms. There was an inter-trial interval of 1500–3000 ms. Participants performed a block of trials where they learned 12 picture–agent pairings. This was followed by a 15 s break and then a new set of 12 picture–agent pairings were presented. Before scanning, participants practiced 18 trials where they were required to learn three picture–agent pairings.

### Computational modelling of behavioural data

Learning behaviour for self, friend and stranger was modelled using an associative learning (AL) algorithm^74^. The AL model has been used in previous studies to examine the behavioural and neural basis of arbitrary visuomotor associations in both non-social and social contexts^16,38,43^. Importantly, although the self-bias has been linked to associative learning processes (e.g. ref. ^7^), associative learning models have not, to our knowledge, been used to understand the mechanisms of ownership, despite providing a powerful tool to define how agent-object association are formed over time. We used this model to examine blood oxygenation-level-dependent (BOLD) signals that scaled parametrically with two parameters, the OAS between picture and agent at the time of the picture, and the size of the OPE at the time of an outcome.

The AL model comprises OAS estimates for self, friend and stranger. On each trial *t*, the OAS estimate for the chosen agent is updated by the choice feedback (correct/incorrect) via a OPE:

1 $${\mathrm{OPE}}_{{\mathrm{chosen Agent}}}\left( {\it{t}} \right){\mathrm{ = Feedback}}\left( t \right)\,{\mathrm{ - }}\,{\mathrm{OAS}}_{{\mathrm{chosen Agent}}}\left( {\it{t}} \right),$$

2 $${\mathrm{OAS}}_{{\mathrm{chosen Agent}}}\left( {{\it{t}} + {\mathrm{1}}} \right) = {\mathrm{OAS}}_{{\mathrm{chosen Agent}}}\left( {t} \right) + {\mathrm{\alpha }} \times {\mathrm{OPE}}_{{\mathrm{chosen Agent}}}\left( {\it{t}} \right).$$

This means that only the OAS of the choice actually made was changed on each trial. The OAS of the remaining two agents were carried over to the next trial unchanged. The learning rate ‘*α*‘ scales the degree to which the prediction error updates the OAS. In our deterministic paradigm, participants are optimal if they have a learning rate closest to ‘1’. The OAS for self, friend and stranger before a new stimulus was encountered for the first time was set to each subject’s average tendency to select self, friend or stranger (see Experimental Task section, Supplementary Note 2 and Supplementary Figure 1 for further details of model selection and comparison).

The probability that a subject makes the choice that is actually observed is calculated via a standard softmax function weighting the OAS of the observed choice by the sum of all three possible choices:

3 $$p({\mathrm{chosen Agent}}) = \frac{{e^{{\mathrm{OAS}}\left( {\mathrm{chosen Agent}} \right)/\beta }}}{{e^{{\mathrm{OAS}}\,\left( {\mathrm{self}} \right)/\beta } + e^{{\mathrm{OAS}}\left( {\mathrm{friend}} \right)/\beta } + e^{{\mathrm{OAS}}\left( {\mathrm{stranger}} \right)/\beta }}}.$$

The temperature parameter ‘*β*’ captures the precision with which the choice is based on the OAS. In addition to the three learning rates, three separate *β* parameters were estimated for self, friend and stranger stimuli (one *β* per true stimulus ownership). Thus, the parameter set *θ* of the model comprised six free parameters. We fitted the model by minimizing the negative log likelihood of the observed choices over all trials *N* individually for each subject:

4 $${\mathrm{nLL = - }}\mathop {\sum}_{t = 1}^{N} {{\mathrm{log(}}{\it{p}}{\mathrm{(chosen Agent}}_{\it{t}}{\mathrm{))}}}.$$

We fitted three separate learning rates for self, friend and stranger OAS updates for several reasons. Based on earlier findings^13^, and previous data showing faster reaction times when making judgements about self-shape pairings compared to stranger-shape pairings^8^, our hypothesis was that these ‘self-biases’ might be explained by people forming associations more rapidly between themselves and objects, and that this would be reflected in a higher learning rate for self. When we examine the model parameters from the separate learning rate parameter model, we can see that this is the case with a main effect of agent learning rate and a significantly higher learning rate for self compared to stranger (Fig. 2e). This three learning rate model also allowed us to explore brain–behaviour correlations in the three learning conditions showing that vmPFC responses for self OAS are correlated with the self learning rate significantly more strongly than vmPFC responses to friend and the friend learning rate and vmPFC responses to stranger and the stranger learning rate. However, when we compared this model to a simpler model with one learning rate and one *β* the simpler model provided a slightly better fit to the data (Supplementary Table 1). To make sure, that differential functional magnetic resonance imaging (fMRI) effects for self and others could not only be driven by differences in learning rates and were not reflective of using a more complex model, we also ran all of our fMRI analyses with the parameter values from the simpler model using a single learning rate for all agents and found no differences in neural responses between the simpler model and our favoured model that allowed us to examine individual differences in behaviour and neural responses. To further assess the suitability of our model, we tested whether our reinforcement learning model explained choices significantly better than chance. First, we calculated the log likelihood of a model in which each choice is made by chance (33% selection probability for each choice). Second, we performed a likelihood ratio test of our model against the chance model^75^. We found that our model indeed explained choices significantly better than the chance model in all our subjects (all *p* < .005; all *χ*^2^(6) > 18.55). We also compared our model to several other plausible control models but our full model still performed better (see Supplementary Note 2 and Supplementary Figure 1).

### Statistical analysis of behavioural data

Analyses of behavioural data were performed in SPSS 24 (IBM Corp, Armonk, NY, USA). We examined differences between conditions in the learning rate at the group level using repeated-measures ANOVAs, with three levels (self, friend and stranger). We examined bivariate associations between the learning rates for self, friend and stranger and neural responses.

### fMRI acquisition and analysis

A Siemens Prisma 3 T MRI scanner was used to acquire multiband T2*-weighted echo planar imaging (EPI) volumes with BOLD contrast. The EPI volumes were acquired in an ascending manner, at an oblique angle (≈30°) to the AC-PC line to decrease the impact of susceptibility artefacts in the orbitofrontal cortex and had the following acquisition parameters: voxel size 2 × 2 × 2, 1 mm gap; TE = 30 ms; repetition time = 1570 ms; flip angle = 90°; field of view = 216 mm. The structural scan was acquired using a magnetization prepared rapid gradient echo sequence with 192 slices; slice thickness = 1 mm; TR = 1900 ms; TE = 3.97 ms; field of view = 192 mm × 192 mm; voxel size = 1 × 1 × 1 mm resolution.

Imaging data were analysed using SPM12 (http://www.fil.ion.ucl.ac.uk/spm). Images were bias corrected then realigned and co-registered to the participant’s own anatomical image. The anatomical image was processed using a unified segmentation procedure combining segmentation, bias correction, and spatial normalization to the MNI template using the New Segment procedure; the same normalization parameters were then used to normalize the EPI images. Lastly, a Gaussian kernel of 8 mm full-width at half-maximum was applied to spatially smooth the images.

Before the study, example first-level design matrices were checked to ensure that estimable GLMs could be performed with independence between the parametric regressors: OAS and OPE for the three types of ownership. This allowed us to look at OAS-related and OPE-related responses independent of one another. The OAS regressors and OPE regressors were also checked to ensure independence at the choice onset and outcome onset. The average correlation between these regressors at the particular time points was <|0.32| (see Supplementary Figure 2) ensuring estimable GLMs.

Four event types were used to construct regressors in which event timings were convolved with SPM’s canonical haemodynamic response function. The onsets of all pictures were modelled with a single regressor and the onsets of the outcomes separated for each agent based on the participants’ choice. These events were modelled as stick functions with 0 duration. Each of these regressors was associated with parametric modulators taken from the computational model. At the time of the picture this was the OAS for self, friend and stranger. Orthogonalisation was turned off and the three parametric modulators were allowed to compete for variance. At the time of the outcome, the ownership PE (calculated as in Eq. 1 above) for self accompanied the outcomes of self choices, friend ownership PE accompanied the outcomes of friend choices and stranger ownership PE accompanied the outcomes of stranger choices. The OAS and OPEs were estimated using average learning rates across the group for each condition to give reliable estimates^75^. In some participants, an eleventh regressor modelled all missed trials, on which participants did not select one of the three agents in the response window. Six head motion parameters modelled the residual effects of head motion as covariates of no interest. Data were high-pass filtered at 128 s to remove low-frequency drifts, and the statistical model included an AR(1) autoregressive function to account for autocorrelations intrinsic to the fMRI time series.

Contrast images from the first level were input into 2 second-level flexible-factorial designs. The first tested for areas that parametrically tracked the OAS at the time of picture presentation (self OAS, friend OAS and stranger OAS) and the second modelled the ownership prediction error at the time of the outcome (self OPE, friend OPE, stranger OPE). All contrasts were examined coding for the linear effect of agent (1 0 −1 and −1 0 1 corresponding to the self, friend and stranger conditions). All common coding analyses were tested using conjunction analysis (AND) to assess significant overlap in neural response. Main effects are reported at *p* < .05, FWE-corrected at the voxel level across the whole brain or *p* < .05 SVC at the voxel level using a combined mask of regions in the mPFC where we had a strong a priori hypothesis and in the subgenual anterior cingulate cortex (ACC) as an additional ROI (see below). We also tested regions that coded OAS and OPEs based on a model with a single learning rate and temperature parameter, to ensure our neural results did not simply reflect the different learning rates for self, friend and stranger. All reported neural results remained the same using parametric values from a two parameter model. We also ran a further GLM that included trial-by-trial reaction times as an additional parametric modulator on the picture period. All reported neural results also remained the same when including reaction time as an additional parametric modulator.

### ROI selection and fMRI contrasts

The a priori ROIs were defined anatomically using masks taken from the atlases of the mPFC in Neubert et al^24^. Mackey and Petrides^23^ have identified similar sub-regions in their analysis of human ventromedial frontal cortical anatomy. We created a combined mask of all our ROIs comprising areas 11 and 14 m (to cover vmPFC), area 9 (to cover dorsomedial prefrontal cortex) and area 24 (to cover the gyral portion of the ACC, extending into dorsal parts of vmPFC). We selected these areas based on previous studies linking responses in these areas to self and/or other^7,8,26,35,37,39^. We also tested whether there were any effects in the subgenual ACC (areas s24 and 25 from the Anatomy Toolbox) as an additional ROI on the basis of a previous study^13^.

### Code availability

Custom Matlab code to implement the learning models is available from the corresponding author upon reasonable request.

### Reporting Summary

Further information on research design is available in the Nature Research Reporting Summary linked to this article.

## Electronic supplementary material

Supplementary Information

Reporting Summary

## Data Availability

The source data underlying all figures is available at: 10.17605/OSF.IO/NWUZ8. Unthresholded statistical parametric maps are available at: https://neurovault.org/collections/4257/. A reporting summary for this article is available as a Supplementary Information file.
