# Supplementary material for: Neural mechanisms for learning self and other ownership
Source: Nat Commun. 2018 Nov 12;9:4747. doi: 10.1038/s41467-018-07231-9 (PMC6232114; doi:10.1038/s41467-018-07231-9)
Supplement: Supplementary file 1 — Supplementary Information [file 41467_2018_7231_MOESM1_ESM.pdf]

Lockwood, Wittmann et al. “Neural mechanisms for learning self and other ownership”

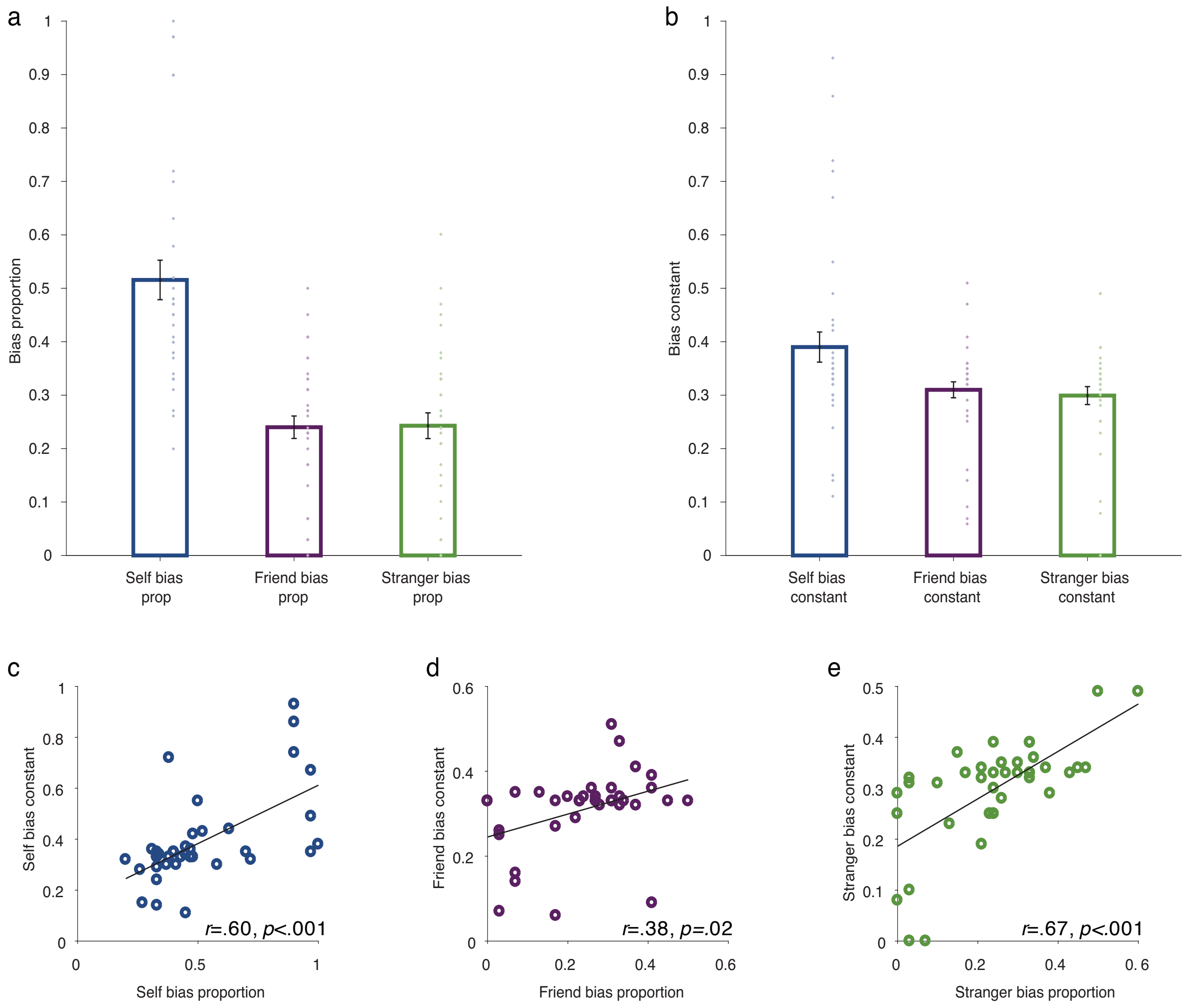

Supplementary Figure 1: Computational models modelling starting bias as starting value vs. free parameter. As detailed in the Methods (section "Computational modelling of behavioural data"), we assessed the initial tendency of participants to choose self, friend, or stranger to new stimuli and used this starting bias (expressed as a choice percentage between 0 and 1 per agent) as the starting value for ownership associative strength (OAS) in our RL model. However, such an initial tendency can also be captured by a free parameter in the model, which we implemented in our control model 'Starting bias as a free parameter' (model 3 in Supplementary table 1; see Supplementary Note 2 for details on how the model was constructed). Panel a shows the distribution of starting values for self, friend and stranger captured by the initial choice percentage, while panel b models the starting bias as a free parameter. Panels c-e show that both variables are significantly positively related for all agents (Pearson correlation, all $p < 0.02$). This suggests that the extra information provided by the additional free parameters was largely redundant to our model-free estimate of the starting bias Error bars shown S.E.M. n=39.

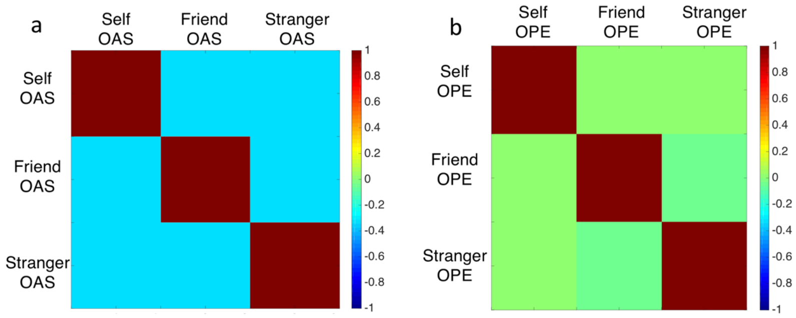

Supplementary Figure 2. Correlations between parametric regressors. (a) correlations between parametric regressors at picture onset (self OAS, friend OAS, stranger OAS). OAS = Ownership Associative Strength at the time of picture onset. (b) correlations between parametric regressors at outcome onset (self OPE, friend OPE, stranger OPE). OPE = Ownership Prediction Error at the time of the outcome. All correlations were below $r<|0.32|$ showing that these regressors could be estimated independently.

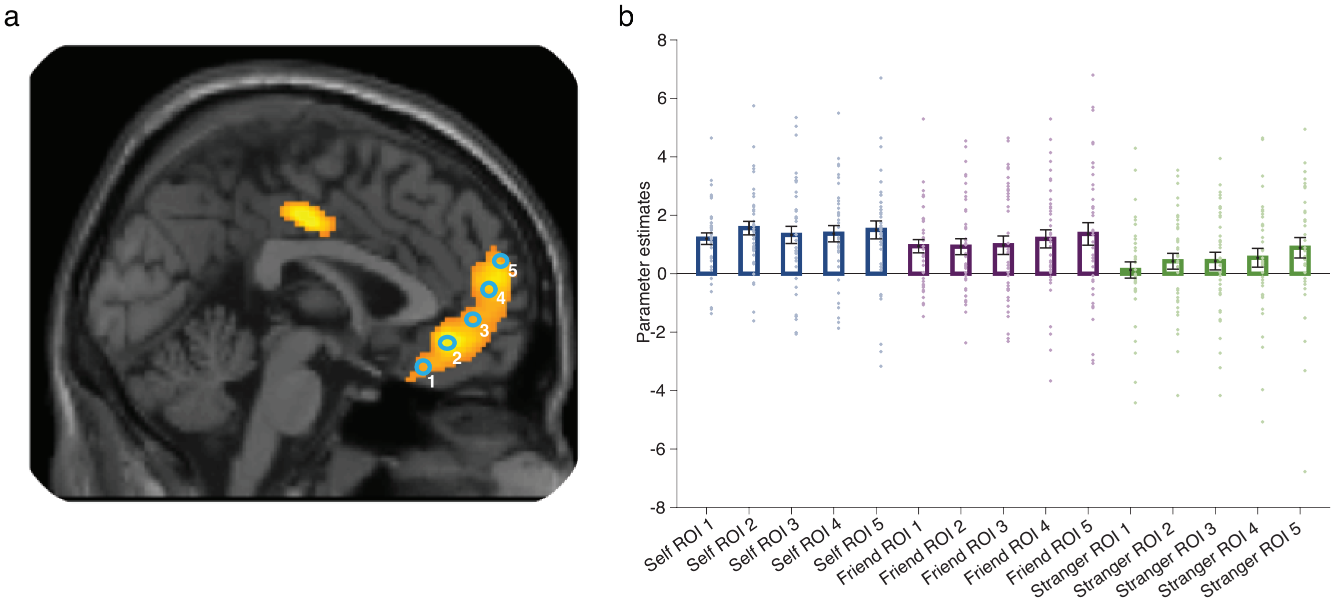

Supplementary Figure 3. Testing a spatial gradient for ownership. (a) Anatomical locations of 5 equally spaced ROIs spanning the ventral to dorsal axis of the main effect of activation to ownership associative strength at the time of choice. Peak ROIs in MNI space were as follows: [x,y,z] = 0,30, -20; 0, 38, -10; 0, 46, 0; 0, 52, 10; 0, 60, 20. (b) Parameter estimates plotted for each agent and each ROI. There was a significant main effect of agent (ANOVA $F(2,76)=3.37$, $p = 0.04$, $\eta^2 = 0.08$) with significantly higher responses across ROIs for self than stranger ($p = 0.02$), a significant main effect of ROI (ANOVA $F(4,152) = 3.01$, $p = 0.02$, $\eta^2 = 0.07$) with higher responses in ROI 5 (dmPFC) than ROI 1 (vmPFC), but no significant agent x ROI interaction (ANOVA $F(8,304) = 0.52$, $p = 0.84$, $\eta^2 = 0.01$). We also tested whether there was a spatial gradient of ownership processing within each agent. This analysis revealed no linear effect of ROI for self (ANOVA $F(1,38) = 0.34$, $p = 0.56$, $\eta^2 = 0.001$) or friend (ANOVA $F(1,38) = 1.62$, $p = 0.21$, $\eta^2 = 0.04$) but intriguingly, a significant linear effect for stranger (ANOVA $F(1,38) = 4.69$, $p < 0.04$, $\eta^2 =.011$) indicating a stronger effect in more dorsal areas. Error bars show S.E.M. N = 39.

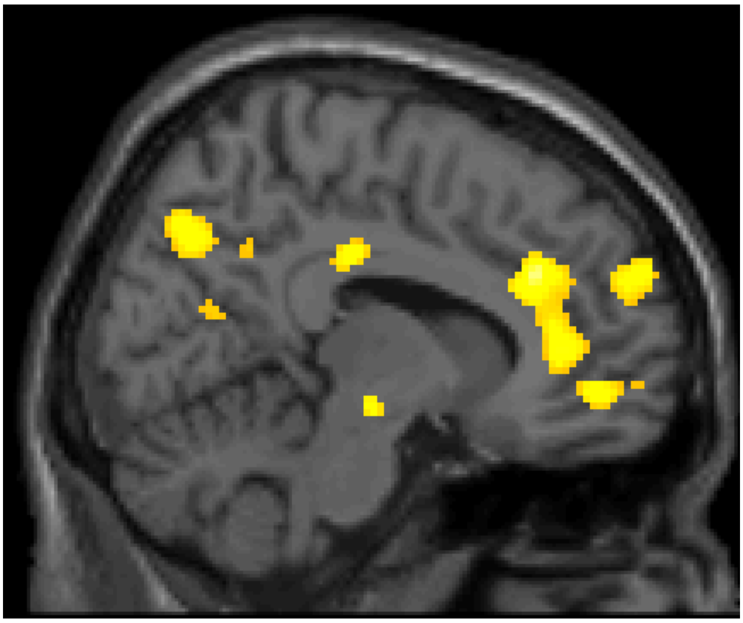

Supplementary Figure 4. Sub-threshold statistical parametric map of neural responses to prediction errors for the contrast "stranger OPE>self OPE". Activation shown on the medial surface overlaid on a single subject T1 scan (*p*<.001 uncorrected). The cluster survives small-volume correction for a combined mask of the medial prefrontal cortex comprising several areas (24, 9, 11m and 14m).

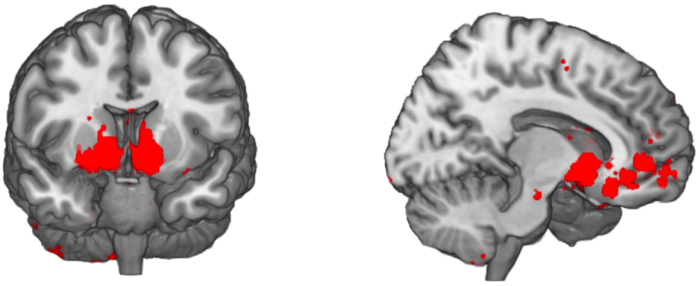

Supplementary Figure 5. **Activation from n=1220 studies of learning and value in Neurosynth.** Combined mask of studies from Neurosynth using key terms ‘value’ and ‘learning’ from n=1220 studies shows significant responses in ventral striatum and vmPFC at standard Neurosynth threshold of FDR 0.01, but no responses in ACCg. Mask overlaid on slice of coronal plane and sagittal plane.

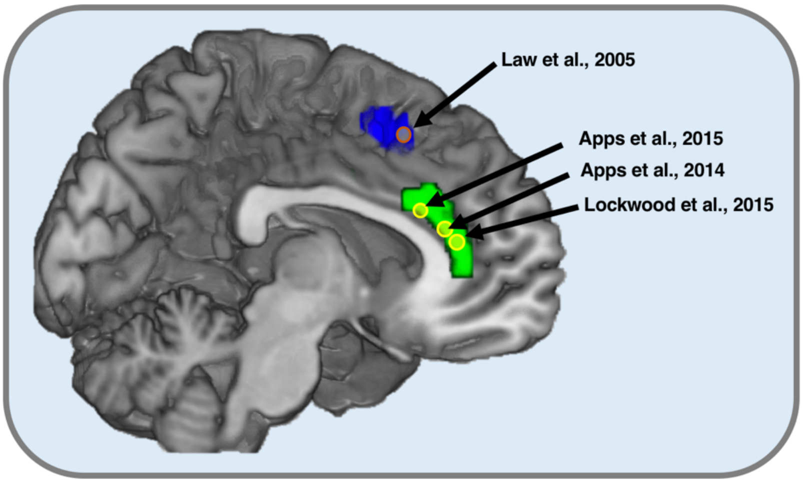

Supplementary Figure 6: Specificity of ACCg for social learning. Peak co-ordinates from studies of socially specific learning[1–3] showing responses along the gyrus of the anterior cingulate cortex (ACCg) and a study of non-social choice-outcome association mappings[4] showing responses in the anterior cingulate sulcus (ACCs) overlaid on an anatomical portion of the medial surface. Green colouring shows activation from our study of ownership prediction errors when learning specifically about strangers and ownership associative strength when tracking friends and strangers. Blue colouring shows domain general responses for ownership associative strength in our study.

Supplementary Table 1

Model	No. of parameters	AIC	NLL
**1) Full model***	**6**	**14333**	**6933**
2) Deterministic model	1	15626	7774
3) Starting bias as a free parameter	8	14600	6988
4) Counterfactual model	7	15218	7336
5) Beta only model	1	14706	7314
**6) One beta and one learning rate***	**2**	**14217**	**7031**

Model comparison. We ran our fmri analyses using both the full model that consisted of 6 parameters (model 1; 3 separate learning rates and 3 temperature parameters) and the simpler model (model 6; one learning rate for all agents and one temperature parameter for all agents), that provided a slightly better fit to the data. Neuroimaging results using both models were similar and all effects that were significant for the more complex full model were also significant using regressors from the simpler model. However, we focused our interpretation on the full model as we observed significant differences in the model learning rate parameters between self and stranger ($p < 0.05$) with a significantly higher learning rate for self, as hypothesised. The full model also allowed us to examine brain-behaviour correlations separately for the different agents. We did however also compare our full model to a range of other plausible control models described in the table. The full model has a better model fit compared to all these control models (model 2-5). See Supplementary Note 2 for a description of the alternative models. For each model, the number of free parameters as well as the model's AIC and NLL are listed. AIC – Akaike Information Criterion. NLL – Negative Log Likelihood.

Supplementary table 2

Conjunction analyses showing overlap in neural responses to ownership associative strength (OAS) and ownership prediction error (OPE). All regions FWE p<.05 voxel-level whole-brain corrected after thresholding at p<.001. BA = Brodmann Area; L – left; R – right; k – cluster extent; ext – extending into.

Brain Region	BA	L/R	Peak voxel			k	t	z
**Conjunction: Ownership Associative Strength (OAS)**								
Inferior frontal gyrus	46	R	52	42	2	762	7.65	6.86
Orbital frontal cortex		R	40	36	-14		5.5	5.17
Supramarginal gyrus	40	L	-62	-48	32	968	5.96	5.55
Superior temporal gyrus	39	L	-42	-56	26		4.62	4.41
		L	-54	-64	22		4.4	4.22
Inferior temporal gyrus *ext. Hippocampus*	20	L	-54	-12	-28	1363	5.91	5.51
		L	-50	0	-34		5.57	5.22
		L	-28	-12	-16		5.28	4.98
Superior Frontal Gyurs	9	R	20	54	30	3671	5.85	5.46
Middle frontal gyrus	10	R	8	58	10		5.52	5.19
		R	12	46	42		5.22	4.94
Posterior cingulate	24	L	-2	-16	40	677	5.55	5.21
		R	16	-20	42		4.4	4.22
		R	14	-10	46		3.69	3.58
Middle temporal gyrus	21	R	60	-44	0	603	5.5	5.17
		R	46	-48	4		4.36	4.18
Supramarginal gyrus	40	R	64	-26	44	616	5.15	4.87
		R	62	-38	46		5.14	4.86
		R	62	-36	36		4.42	4.24
Middle temporal gyrus	21	R	56	-18	-12	773	4.99	4.74
		R	56	6	-30		4.8	4.57
		R	50	-2	-32		4.65	4.44
**Conjunction: Ownership Prediction Error (OPE)**								
Ventral striatum *ext. Oribital Frontal Cortex*		L	-14	10	-8	959	7.23	6.54
Ventral striatum		L	-14	2	-14		6.83	6.24
		L	-22	26	-18		3.81	3.69
Ventral striatum		R	16	6	-12	554	6.99	6.37
Superior frontal gyrus	8	L	-18	30	56	404	4.99	4.73

Supplementary Note 1

Self bias at start does not account for the faster reaction times and higher accuracy for self ownership. As explained in the main text, subjects exhibited a bias at the start of the task to indicate that stimuli were owned by self as opposed to the friend or stranger, and we observed a faster learning rate for self ownership compared with stranger ownership. It is therefore possible that faster self-related learning might result from participants learning more quickly from confirmatory feedback experienced after a self ownership response. We therefore performed an additional control analysis to examine whether the faster learning rate for self ownership was simply a consequence of the starting bias to indicate self ownership. To do this we examined differences in reaction times and accuracy in a subset of participants and trials where participants did not start with labelling the stimuli correctly as 'self' on the first trial (or correctly as friend for friend stimuli or stranger for stranger stimuli). We included participants who failed to respond correctly to at least two out of each set of eight stimuli (for each set of self-, friend-, and stranger-related stimuli) on the first trial. This left a sample of n = 31. We focused on our main comparisons of *stranger RT >self RT* and *self correct >stranger correct* due to the reduction in power from including fewer participants and a subset of trials. We found that the comparison of faster reaction times for self compared to stranger remained significant ($t(30) = -3.53$, $p<.001$) as did the results for accuracy ($t(30) = 2.13$, $p<.05$), even after accounting for the starting bias.

We note in addition that the analysis of stay/switch is conducted on the tendency to stay/switch at trial t+1 and therefore is not subject to the starting bias. In our learning rate analyses we used the average tendency to select self, friend, or stranger on the first trial as a starting value (see Methods). Overall, we show distinct effects of self-ownership processing in initial biases, reaction times, accuracy, stay/switch, and learning rates.

Supplementary note 2

Control models. We also compared our associative learning model to another set of models that may also describe how people learn in our task. See Supplementary Table 1 for a summary. The first model, termed the 'deterministic model' (model 2 in Supplementary Table 1) assumes that learning that a fractal does or does not belong to one of the agents has consequences for the OAS for the other agents. Moreover, this model assumes that this update process is asymmetrical for correct compared to incorrect choices depending on the amount of exposure to a stimulus. This

model uses just a single free parameter, which is the beta weight of the softmax decision function. The softmax function is otherwise identical to the softmax function of our full model (the same goes for all other alternative models unless otherwise noted). On the first trial of each stimulus, OASs are still modelled based on the starting bias (same as in the full model). However, we then assumed that subjects might engage in deductive reasoning exploiting the deterministic nature of the task. Thus, OAS are not updated via a delta rule but instead based on the logical contingencies of the task. This was done in the following way: When people were correct on the first trial, the model set the OAS of the chosen agent to 1 and the OAS of both unchosen agents to zero, reflecting that subjects knew whom the stimulus did and did not belong with a 100% certainty. When people instead were incorrect once, the model assumed that people know that the cue does not belong to the chosen agent but might belong to the other two agents with equal probability (given that none of them had been chosen already). Accordingly, the chosen agent's OAS was set to 0 and the two unchosen agents' OAS were set to 0.5. After two negative feedbacks for two different agents, the true identity of the owner can also be discerned with 100% certainty, so for this case the OAS for the correct agent was set to 1 and for the two incorrect agents to 0. Once the correct owner could be logically identified, the OAS of the agents remained unchanged. Comparing the fit of this deterministic model to our full model showed a better fit for our full model (full model AIC = 14333, deterministic model AIC = 15626) suggesting that participants exhibit learning closer to our full model.

Next, the ‘starting bias as a free parameter’ model assumes the starting bias can be modelled as a free parameter rather than as a starting value as in the full model (see Supplementary Fig.1; model 3 in Supplementary Table 1.). We built a modified version of our model in which we did exactly that. For the starting bias to reflect a choice probability between 0 and 1, the starting biases for self, friend and stranger need to add up to 1. Hence, we fitted two additional free parameters for self and friend and defined the third one (stranger) as "1-startingBias(self)-startingBias(friend)". We compared this new model with our RL model using Bayesian Model Comparison (Akaike Information Criterion). Our model using the participants’ proportions of self, friend, and stranger response on the first choice provided a better fit to the data than a model treating the starting bias as a free parameter. However, the estimates of bias from the two models were significantly correlated for all agents indicating that the additional information provided by the additional free parameters was largely redundant to our model-free estimate of the starting bias (Supplementary Figure 1).

The counterfactual model (model 4, Supplementary Table 1) that is the same as the full model but presumes in addition participants also engage in counterfactual learning and therefore a counterfactual learning rate reflects the degree to which unchosen options are updated inversely to the chosen option. We modelled a single counterfactual learning rate that was used independent of which agent was chosen. The prediction error for the chosen agent was sign-reversed and added to both unchosen options scaled by the counterfactual learning rate.
Finally, we also compared our original model to a model that assumed that people have a learning rate of 1 for all agents. We used only a single beta weight as a free parameter for this model and no other free parameters (Beta only model; model 5 in Supplementary Table 1). This model is similar to the deterministic model in assuming people update completely from 1 trial, but more similar to our original 3 learning rate model. This 'Beta only model' also performed worse than our full model (full model AIC = 14333, 1LR model AIC = 14706).

Supplementary note 3

Ventromedial prefrontal cortex activity and the learning rate. Since we found that there were significant correlations only between self learning rate and self OAS coding in vmPFC but no significant associations with friend and stranger learning rates and associated vmPFC responses we tested whether the correlations between vmPFC and the self-associated learning rate were significantly higher than correlations between vmPFC responses to friend and friend learning rate and vmPFC responses to stranger and stranger learning rate. Given our directional hypothesis we focused on one-tailed tests.

We found a significantly stronger correlation between the self-associative learning rate and vmPFC response to self OAS, than friend-associative learning rate and vmPFC responses to friend OAS (Pearson-Filon $Z = 2.04$, $p = 0.02$ one-tailed). We also observed significantly stronger correlations with self-associative learning rate and self OAS in vmPFC, compared to stranger-associative learning rate and vmPFC responses to stranger OAS (Pearson-Filon $Z = 1.70$, $p = 0.04$ one-tailed). This suggests that increased vmPFC responses to self OAS vs. stranger OAS and friend OAS did not only reflect the increased learning rate for self, but also a more fundamental ownership bias for forming associations between objects and the self as opposed to any other agent.

A key component of the associative learning model is that prediction errors are scaled by learning rates[5] and prediction error-related activity has been reported in vmPFC[6,7]. In line with previous

findings we also observed that average response in the same vmPFC area that responded to self>stranger OAS also significantly tracked self OPEs (t=3.32, p<0.001), friend OPEs (t= 2.86, p=0.002) and stranger OPEs (t=4.86, p<.001). We therefore tested whether the average response in the same vmPFC region also had OPE-related activity that correlated most strongly with self-associative learning rates.

We found a significant association between the self-associative learning rate and vmPFC responses to self OPEs (r = 0.38, p = 0.02, 95% CI [.07, .62]). There was no significant correlation between vmPFC responses to friend OPEs and friend-ownership learning rates (r = 0.16, p = 0.33, 95% CI [-.16, .45]), or vmPFC responses to stranger OPEs and stranger-ownership learning rates (r = -0.06, p = 0.72, 95% CI [-.37, .26]). The self-ownership learning rate and prediction error correlations were significantly stronger than the correlations pertaining to other agents that were strangers (Pearson-Filon Z = 2.19, p = 0.01 one-tailed) but not other agents that were friends (Pearson-Filon Z = 1.13, p = 0.13 one-tailed).

Supplementary note 4

Possible gradients in response properties across medial frontal cortex. A final plausible hypothesis for how the brain may reflect self and other relevant processing is based on a gradient within the medial prefrontal cortex with self-related responses in ventral parts of mPFC transitioning to other-related processing in dmPFC[8]. To test this hypothesis we selected 5 equally spaced peak ROIs spanning the ventral to dorsal axis of our activation for the main effect (contrast 1,1,1) of ownership associative strength (OAS) at the time of the picture (thresholded at p < 0.05 FWE whole brain corrected to ensure activation was specifically on the medial surface, see supplementary Figure 3a). We then conducted a 5 (ROI) by 3 (self, friend, stranger) ANOVA that showed a main effect of agent, a main effect of ROI but crucially no significant agent x ROI interaction (F(8,304) = 0.52, p = 0.84, η^2 = 0.01). Intriguingly, when testing for a linear effect in each agent separately we did observe a spatial gradient for stranger ownership associations with highest responses in dorsal compared to ventral areas, but not for self or friend (see Supplementary Figure 3b). Our analysis suggests, once again, that when it comes to basic ownership associations encoding of self-related associations predominates throughout both dmPFC and vmPFC. There was, however, evidence for the encoding of other related associations throughout mPFC and there was tentative evidence that this encoding became stronger moving from vmPFC to dmPFC. It would be interesting in future studies to dissect the different conditions in which a self-other gradient arises.

Supplementary note 5

Meta-analysis of spatial location of value and learning related activity. To test whether the areas we observed for learning about ownership were similar to areas that track domain general responses to value and learning we conducted a reverse inference meta-analysis using Neurosynth, an online tool to generate probabilistic mappings between cognitive and neural states across large numbers of studies[9]. We ran meta-analyses using separate keywords of 'value' and 'learning' to create a combined mask comprising n=1220 studies (Supplementary Figure 5). We then tested this mask in all of our contrasts and observed significant responses in ventral striatum from the meta-analysis that overlapped with a domain-general (self, friend, stranger) response to ownership associative strength and ownership prediction errors. We found significant responses in ventral striatum and vmPFC ($p < 0.05$ SVC for the neurosynth mask) that overlapped with the neurosynth mask. We did not observe any significant responses in ACCg, the area we found to be specific for social learning in our task.

References

1. Apps, M. A. J. & Ramnani, N. The anterior cingulate gyrus signals the net value of others' rewards. *J. Neurosci.* **34,** 6190–6200 (2014).
2. Apps, M. A. J., Lesage, E. & Ramnani, N. Vicarious reinforcement learning signals when instructing others. *J. Neurosci.* **35,** 2904–2913 (2015).
3. Lockwood, P. L., Apps, M. A. J., Roiser, J. P. & Viding, E. Encoding of Vicarious Reward Prediction in Anterior Cingulate Cortex and Relationship with Trait Empathy. *J. Neurosci.* **35,** 13720–13727 (2015).
4. Law, J. R. *et al.* Functional magnetic resonance imaging activity during the gradual acquisition and expression of paired-associate memory. *J. Neurosci. Off. J. Soc. Neurosci.* **25,** 5720–5729 (2005).
5. Sutton, R. S. & Barto, A. G. *Reinforcement learning: an introduction.* (MIT press, 1998).

6. Rutledge, R. B., Dean, M., Caplin, A. & Glimcher, P. W. Testing the reward prediction error hypothesis with an axiomatic model. *J. Neurosci. Off. J. Soc. Neurosci.* **30,** 13525–13536 (2010).
7. Suzuki, S. *et al.* Learning to simulate others' decisions. *Neuron* **74,** 1125–37 (2012).
8. Sul, S. *et al.* Spatial gradient in value representation along the medial prefrontal cortex reflects individual differences in prosociality. *Proc. Natl. Acad. Sci.* **112,** 7851–7856 (2015).
9. Yarkoni, T., Poldrack, R. A., Nichols, T. E., Van Essen, D. C. & Wager, T. D. Large-scale automated synthesis of human functional neuroimaging data. *Nat. Methods* **8,** 665–670 (2011).
