# Supplementary material for: Neural mechanisms for learning self and other ownership
Source: Nat Commun. 2018 Nov 12;9:4747. doi: 10.1038/s41467-018-07231-9 (PMC6232114; doi:10.1038/s41467-018-07231-9)
Supplement: Supplementary file 2 — Reporting Summary [file 41467_2018_7231_MOESM2_ESM.pdf]

nature research

Corresponding author(s): Patricia Lockwood

Reporting Summary

Nature Research wishes to improve the reproducibility of the work that we publish. This form provides structure for consistency and transparency in reporting. For further information on Nature Research policies, see Authors & Referees and the Editorial Policy Checklist.

Statistical parameters

When statistical analyses are reported, confirm that the following items are present in the relevant location (e.g. figure legend, table legend, main text, or Methods section).

n/a	Confirmed	
☐	☒	The exact sample size (*n*) for each experimental group/condition, given as a discrete number and unit of measurement
☐	☒	An indication of whether measurements were taken from distinct samples or whether the same sample was measured repeatedly
☐	☒	The statistical test(s) used AND whether they are one- or two-sided<br>*Only common tests should be described solely by name; describe more complex techniques in the Methods section.*
☒	☐	A description of all covariates tested
☐	☒	A description of any assumptions or corrections, such as tests of normality and adjustment for multiple comparisons
☐	☒	A full description of the statistics including central tendency (e.g. means) or other basic estimates (e.g. regression coefficient) AND variation (e.g. standard deviation) or associated estimates of uncertainty (e.g. confidence intervals)
☐	☒	For null hypothesis testing, the test statistic (e.g. *F*, *t*, *r*) with confidence intervals, effect sizes, degrees of freedom and *P* value noted<br>*Give P values as exact values whenever suitable.*
☒	☐	For Bayesian analysis, information on the choice of priors and Markov chain Monte Carlo settings
☒	☐	For hierarchical and complex designs, identification of the appropriate level for tests and full reporting of outcomes
☐	☒	Estimates of effect sizes (e.g. Cohen's *d*, Pearson's *r*), indicating how they were calculated
☐	☒	Clearly defined error bars<br>*State explicitly what error bars represent (e.g. SD, SE, CI)*

Our web collection on statistics for biologists may be useful.

Software and code

Policy information about availability of computer code

Data collection	Presentation (Neurobehavioural systems) was used to collect the data.
Data analysis	Data analysis was conducted in SPSS version 25 and Matlab 2015b

For manuscripts utilizing custom algorithms or software that are central to the research but not yet described in published literature, software must be made available to editors/reviewers upon request. We strongly encourage code deposition in a community repository (e.g. GitHub). See the Nature Research guidelines for submitting code & software for further information.

Data

Policy information about availability of data

All manuscripts must include a data availability statement. This statement should provide the following information, where applicable:

- Accession codes, unique identifiers, or web links for publicly available datasets
- A list of figures that have associated raw data
- A description of any restrictions on data availability

Data used to generate all figures is available at . Unthresholded T maps of the relevant data used for all fmri contrasts is available at Neurovault (https://neurovault.org/collections/4257/)

Field-specific reporting

Please select the best fit for your research. If you are not sure, read the appropriate sections before making your selection.

☐ Life sciences ☒ Behavioural & social sciences ☐ Ecological, evolutionary & environmental sciences

For a reference copy of the document with all sections, see nature.com/authors/policies/ReportingSummary-flat.pdf

Behavioural & social sciences study design

All studies must disclose on these points even when the disclosure is negative.

Study description	Data are quantitative experimental data.
Research sample	A convenience sample of students, staff and public members in the vicinity of Oxford took part in the study.
Sampling strategy	Random sampling was used. We conducted an a priori power calculation based on our planned sample size and desired power (80% at alpha p=0.05) to show that with 39 subjects we had 80% power to detect a 'medium' effect size of d = 0.46 at alpha = 0.05 (two-tailed) in any of our behavioural measures, an effect size smaller than typically reported in this field, indicating sufficient power.
Data collection	Participants performed the task inside an MRI scanner in a separate room to the researcher and radiogrpahers.
Timing	Data collection took place between July 2016 and September 2016.
Data exclusions	One participant was excluded from the analysis due to a neurological abnormality identified during MRI scanning.
Non-participation	No participants dropped out or declined participation.
Randomization	There was only one experimental group.

Reporting for specific materials, systems and methods

Materials & experimental systems

n/a	Involved in the study	
☒	☐	Unique biological materials
☒	☐	Antibodies
☒	☐	Eukaryotic cell lines
☒	☐	Palaeontology
☒	☐	Animals and other organisms
☐	☒	Human research participants

Methods

n/a	Involved in the study	
☒	☐	ChIP-seq
☒	☐	Flow cytometry
☐	☒	MRI-based neuroimaging

Human research participants

Policy information about studies involving human research participants

Population characteristics	See above.
Recruitment	Participants were recruited through the departmental database, emails to students and word of mouth.

Magnetic resonance imaging

Experimental design

Design type	Event-related design.
Design specifications	Participants completed 246 trials in total. 126 in the first block and 120 in the second block. Each trial lasted approximately 8.7 seconds with a variable jitter. See Figure 1 for full details of the timing of different experimental events.

Behavioral performance measures: Correct button presses and response times were recorded. An ANOVA was used to determine that there was a significant main effect of stimulus repetition, showing that participants were able to learn throughout the experiment.

Acquisition

Imaging type(s): Functional and structural.

Field strength: 3 Tesla.

Sequence & imaging parameters: A Siemens Prisma 3T MRI scanner was used to acquire multiband T2*-weighted echo planar imaging (EPI) volumes with blood oxygenation-level–dependent (BOLD) contrast. The EPI volumes were acquired in an ascending manner, at an oblique angle (≈30˚) to the AC-PC line to decrease the impact of susceptibility artefacts in the orbitofrontal cortex and had the following acquisition parameters: voxel size 2 x 2 x 2, 1 mm gap; TE=30 ms; repetition time=1570ms; flip angle=90°; field of view=216 mm.

Area of acquisition: Whole brain scan.

Diffusion MRI ☐ Used ☒ Not used

Preprocessing

Preprocessing software: SPM 12 was used to preprocess the data.

Normalization: The data were normalised using the standard New Segment procedure implemented in SPM12.

Normalization template: The MNI template was used as is standard in SPM.

Noise and artifact removal: 6 realignment parameters were included in the design matrices.

Volume censoring: No volume censoring was used. Participant motion was evaluated by examining scans where there was movement greater than 1mm in any direction. Flagged scans were visually inspected. No participant had more than 4% of scans affected by motion and no flagged scans showed visible motion artifacts.

Statistical modeling & inference

Model type and settings: We used univariate models. Contrast images from the first level were input into two second-level flexible-factorial designs. The first tested for areas that parametrically tracked the OAS at the time of picture presentation (Self OAS, Friend OAS, Stranger OAS) and the second modelled the ownership prediction error at the time of the outcome (Self OPE, Friend OPE, Stranger OPE).

Effect(s) tested: The first tested for areas that parametrically tracked the OAS at the time of picture presentation (Self OAS, Friend OAS, Stranger OAS) and the second modelled the ownership prediction error at the time of the outcome (Self OPE, Friend OPE, Stranger OPE). Second-level flexible factorial models were used.

Specify type of analysis: ☐ Whole brain ☐ ROI-based ☒ Both

Anatomical location(s): The a priori regions of interest (ROIs) were defined anatomically using masks taken from the atlases of the medial prefrontal cortex in Neubert and colleagues (2013). Mackey and Petrides have identified similar sub-regions in their analysis of human ventromedial frontal cortical anatomy (2014). We created a combined mask of all our ROIs comprising areas 11m and 14m (to cover vmPFC), area 9 (to cover dorsomedial prefrontal cortex) and area 24 (to cover the gyral portion of the anterior cingulate cortex, extending into dorsal parts of vmPFC). We selected these areas based on previous studies linking responses in these areas to self and/or other (Sui et al., 2015; Sui et al., 2013; Kelley et al., 2002; Witmann et al., 2018; Wittmann et al., 2016; Apps et al., 2016). We also tested whether there were any effects in the subgenual anterior cingulate cortex (areas s24 and 25 from the Anatomy Toolbox) as an additional ROI on the basis of a previous study (Lockwood et al., 2016).

Statistic type for inference (See Eklund et al. 2016): Voxel-wise family-wise error correction was used.

Correction: Family wise error correction (p<.05) and small volume family wise error correction in predetermined regions of interest (p<.05 SVC).

Models & analysis

n/a	Involved in the study
☒	☐ Functional and/or effective connectivity
☒	☐ Graph analysis
☒	☐ Multivariate modeling or predictive analysis
